# Pharmacological evidence for lithium-induced neuroprotection against methamphetamine-induced neurodegeneration via Akt-1/GSK3 and CREB-BDNF signaling pathways 

**DOI:** 10.22038/ijbms.2019.30855.7442

**Published:** 2019-08

**Authors:** Shafagh Mehrafza, Sareh Kermanshahi, Shahnaz Mostafidi, Majid Motaghinejad, Manijeh Motevalian, Sulail Fatima

**Affiliations:** 1 Department of Pharmaceutical Chemistry, Faculty of Pharmaceutical Chemistry, Pharmaceutical Sciences Branch, Islamic Azad University (IUAPS), Tehran, Iran; 2 Research Center for Addiction and Risky Behaviors (ReCARB), Iran Psychiatric Center, Iran University of Medical Sciences, Tehran, Iran; 3 Department of Pharmacology, School of Medicine, Iran University of Medical Sciences, Tehran, Iran; 4 Department of Physiology, Tehran University of Medical Sciences-International Campus (TUMS-IC), Tehran, Iran

**Keywords:** Akt, BDNF, CREB, Lithium, Methamphetamine, GSK3

## Abstract

**Objective(s):**

Neurodegeneration is an outcome of Methamphetamine (METH) abuse. Studies have emphasized on the neuroprotective properties of lithium. The current study is designed towards evaluating the role of Akt-1/GSK3 and CREB-BDNF signaling pathways in mediating lithium neuroprotection against METH-induced neurodegeneration in rats.

**Materials and Methods:**

Sixty adult male rats were randomly divided into five groups: control group (received 0.7 ml normal saline per rat for 28 days), METH group (given 10 mg/kg of METH intraperitoneally for 28 days), groups 3, 4, and 5 (given METH (10 mg/kg) and lithium (75, 150, and 300 mg/kg intraperitoneally, individually for 28 days). Morris water maze (MWM) was used to assess mental functions. In addition to hippocampal neurodegeneration, Brain-derived neurotrophic factor (BDNF), cAMP response element binding (CREB), Glycogen synthase kinase 3 (GSK3), and Protein kinase B (Akt-1) were assessed in isolated hippocampus.

**Results:**

METH abuse caused marked disorders in learning and memory that were dramatically improved with various doses of lithium. Furthermore, METH increased lipid peroxidation and the levels of oxidized form of interleukin 1 beta (IL-1β), glutathione (GSSG), Bax, tumor necrosis factor alpha (TNF-α), and GSK3, while attenuating the extent of glutathione (reduced form (GSH)), P-CREB, Bcl-2, BDNF, and Akt-1 in the hippocampus. Moreover, METH declined superoxide dismutase (SOD), glutathione reductase (GR), and glutathione peroxidase (GPx) activity in the hippocampus. Conversely, lithium attenuated METH-stimulated apoptosis, oxidative stress, and inflammation; while improving the extent of BDNF and P-CREB.

**Conclusion:**

Probably lithium possesses neuroprotection against METH-stimulated neurodegeneration in the hippocampus via Akt-1/GSK3β and CREB/BDNF signaling pathways.

## Introduction

Methamphetamine (METH), a neurostimulator agent, is widely misused all over the world. The concerns of prolonged use of METH and its biochemical and behavioral effects are still imprecise (1-4). The release of norepinephrine, dopamine, and to a lesser degree serotonin into synaptic terminals is increased via METH. Furthermore, the consequences of METH abuse are hyper-activation of dopaminergic receptors in the acute phases and down-regulation of these receptors in the chronic phase (4, 5). The high risk of addiction and misuse of METH is caused by similarity of its biological action, compared to cocaine (5). According to investigations on experimental rodent models, chronic abuse of METH and its derivatives can prompt behavioral changes such as mood disorder and cognitive (learning and memory) damage (6-8). Also, studies have established the potential effect of METH in neurodegeneration of some brain areas such as the hippocampus, which is intensely connected with cognitive processes (7). Moreover, previous studies have displayed that METH abuse can stimulate apoptotic pathways and consequently, cause DNA fragmentation in brain cells (9, 10). It is well-known that METH and other neuro stimulants can prompt inflammation, mitochondrial malfunction, and oxidative stress in brain cells, though, the supposed mechanism are still unknown (11, 12). The Food and Drug Administration (FDA) has approved lithium as the first drug for maintenance therapy of bipolar disorder. Its actions of mood stabilization and manic and depressive prevention are regulated by calcium homeostasis, electrical excitation, neurotransmission, arachidonic acid metabolism, protein kinase C, and adenylyl cyclase system signaling (13). Furthermore, lithium impedes glycogen synthase kinase-3β (GSK-3), an enzyme associated with the inhibition of several cell survival factors such as cyclic AMP response element binding protein (CREB) and β-catenin (14-18). CREB regulates genes related with survival of neurons, neuroprotection, and neural plasticity such as brain-derived neurotrophic factor (BDNF) (19). Other than its anti-apoptotic action, lithium carries profound antioxidant, anti-inflammatory (14, 20), and neuroprotective potential (14, 20, 21). It has also been shown to reduce the drug abuse-stimulated increase in IL-1β, TNF-α, and apoptotic factor levels (20), through chronic treatment. Lithium-induced cognitive enhancements are also emphasized by human and animal-based studies (22). It is proposed that lithium may protect hippocampal neurons against METH-stimulated oxidative stress, apoptosis, and inflammation impairment through up-regulation of BDNF and CREB and regulation of Akt-1 and GSK3, but this mechanism has not been ascertained definitely. Given the importance of Akt-1/GSK3 and P-CREB/BDNF signaling pathways in conferring cognitive functions and neuroprotection, we designed this study to evaluate the role of these paths intermediating lithium-induced neuroprotection in contrast to methamphetamine stimulated inflammation, oxidative stress, apoptosis, and cognitive disturbances. This study is of specific status as it offers a vision into the disorders related to METH abuse and neuroprotective action of lithium against such disturbances. 

## Materials and Methods

***Animals***

Sixty adult Wistar male rats, weighing 250–300 g, were attained from the Experimental Research Center (Iran University of Medical Sciences). All animals were housed under controlled conditions with 22 ± 0.5 °C; 12-hr light/dark rotation and had access to food and water *ad libitum*. The experimental procedure was approved by the Research Council of Iran University of Medical Sciences, Tehran, Iran (This research is supplementary data from PhD thesis with code number: 14 and Research Code:6237). The experiment was planned as stated in ARRIVE (Animal Research: Reporting of In vivo Experiments) guiding principle, and all technical and ethical aspects were taken into account (23, 24).

***Drug***

*Drugs*

Lithium and methamphetamine were obtained from the Sigma-Aldrich company and used as soon as prepared.

***Experimental design***

Group 1 (control) received normal saline (0.7 ml/rat, IP) for 28 days.

Group 2 (methamphetamine) received methamphetamine (10 mg/kg, IP) for 28 days.

Groups 3, 4, and 5 received methamphetamine (10 mg/kg, IP) and lithium (75, 150, and 300 mg/kg, IP separately) for 28 days, simultaneously. 

The Morris Water Maze (MWM) test was done, during days 24 to 28, for evaluation of the effect of methamphetamine alone or in combination with lithium on memory and spatial learning. Once cognitive activity was evaluated (on day 28) and 24 hr after last administration of the drug, 50 mg/kg of sodium thiopental was intraperitoneally administrated for anaesthetization of rats. Afterward, total hippocampus was separated in order to detect bio-indicators of apoptosis, inflammation, and oxidative stress. The molecular and behavioral tests were done by the experimenter blinded to the animals’ identity. 

***Cognitive assessment***

*Morris water maze (MWM) experiment*

The MWM apparatus, including a black round tank (160 x 90 cm) filled with water, was placed in the center of the experimental laboratory. This equipment was separated into four quadrants (North, East, West, and South) and water filled to 50 cm height. The blind experimenter waited in the North-East area of the room. A stand, with a length of 15 cm, was hidden and placed 1 cm below the water surface. During the training procedure (first four days), the mentioned stand was located randomly in one of the quadrants. A tracking system, automated with an infrared camera (CCTV B/W, SBC-300 (P), Samsung Electronics Co, Ltd, and Korea) was used to assess animal location in the tank. The camera was located 2.4 m above the water surface (25, 26).

**Figure 1:**
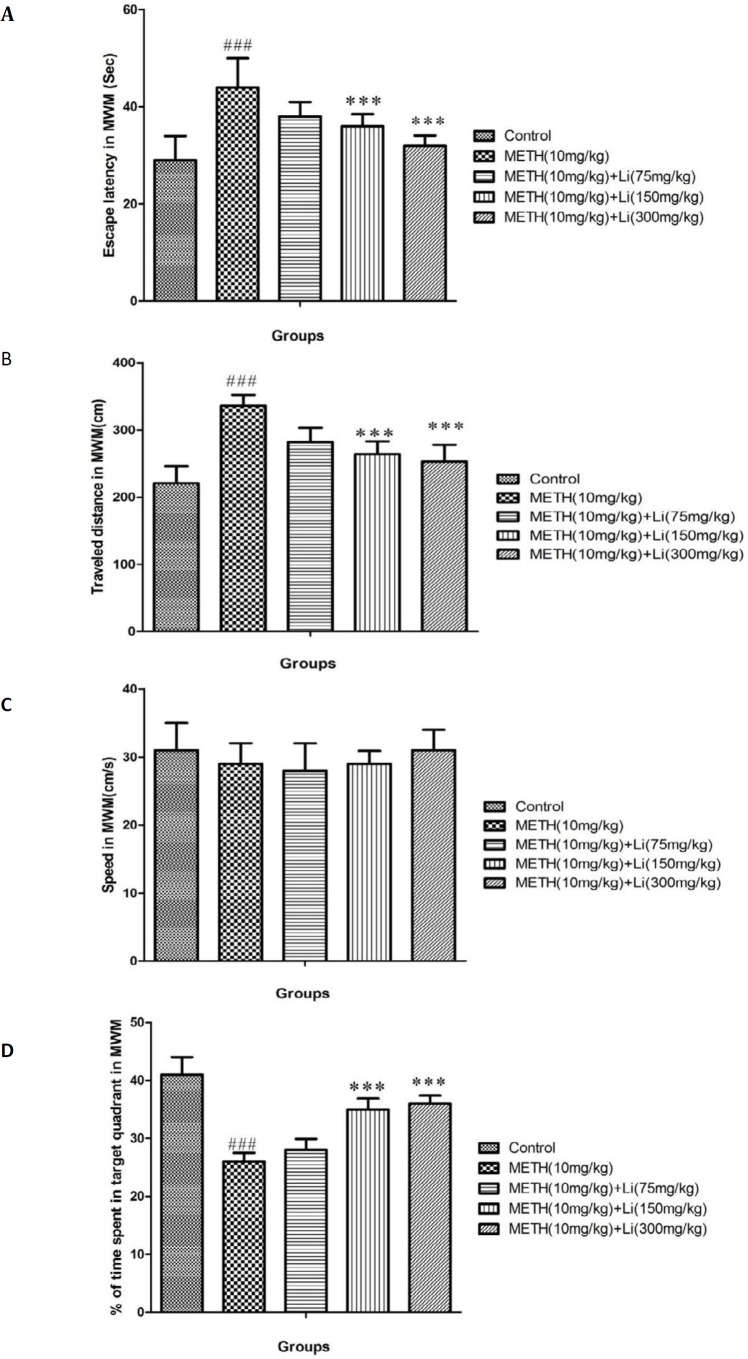
Learning and memory assessment by using Morris Water Maze (MWM). (A) average escape latency, (B) average distance traveled, (C) average swimming speed, and (D) percentage of time spent in target quadrant in probe trial in control group and groups treated with 10 mg/kg of methamphetamine and 10 mg/kg of methamphetamine in combination with lithium with doses of 75, 150, and 300 mg/kg across all training

All data are expressed as mean ± SEM (n=8)

### *P*<0.001 vs control; *** *P*<0.001 vs 10 mg/kg of methamphetamine

METH: Methamphetamine; Li: Lithium

**Figure 2:**
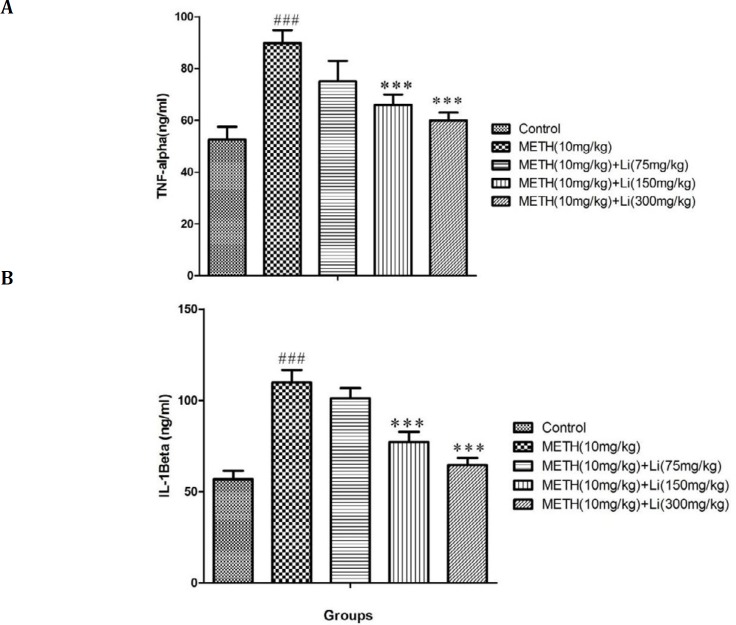
Effects of various doses of lithium (75, 150, and 300 mg/kg) on methamphetamine-induced s in (A) TNF-α and (B) IL-1β level in rat isolated hippocampus. All data are expressed as mean ± SEM (n=8). ### *P*<0.001 vs control; *** *P*<0.001 vs 10 mg/kg of methamphetamine

METH: Methamphetamine. Li: Lithium

**Figure 3:**
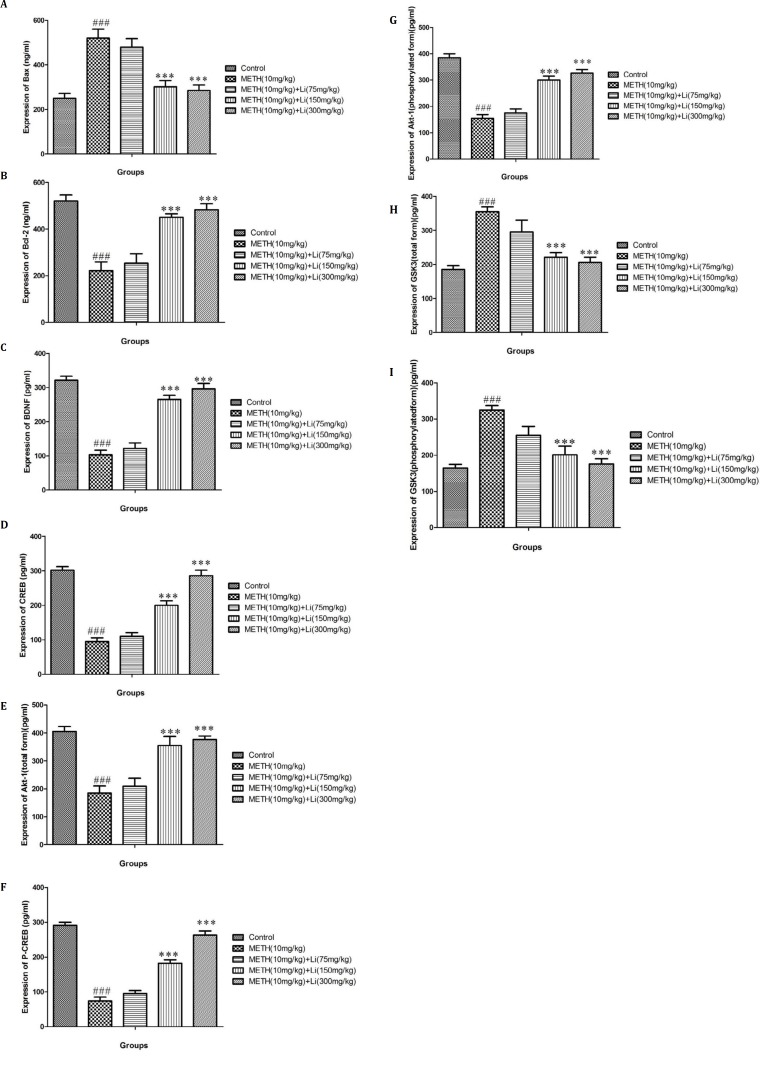
Effects of various doses of lithium (75, 150, and 300 mg/kg) on methamphetamine-induced alterations in protein expression of (A) Bax, (B) Bcl-2, (C) BDNF total, (D) CREB, (E) phosphorylated CREB , (F) total Akt-1, (G) phosphorylated Akt-1, (H) total GSK3, and (I) phosphorylated GSK3 in rat isolated hippocampus

All data are expressed as mean±SEM (n=8)

### *P*<0.001 vs control

*** *P*<0.001 vs 10 mg/kg of methamphetamine

METH: Methamphetamine; Li: Lithium

**Table -1:** Effects of various doses of lithium on methamphetamine-induced GSH/GSSG alterations in hippocampal mitochondria

**Group **	**GSH (** **nmol/mg of protein)**	**GSSG (nmol/mg of protein)**	** GSH/GSSG**
**Control**	65.2±5.1	0.82±0.2	79
**METH (10 mg/kg)**	38.5±3.1^a^	4.3±1.7^a^	8.8^a^
**METH (10 mg/kg) +Li (75 mg/kg)**	44.5±3.5	3±0.09	19
**METH (10 mg/kg) +Li (150 mg/kg)**	50.5±3 b	1.93±0.13^ b^	25^b^
**METH (10 mg/kg) +Li (300 mg/kg)**	54.6±3.8 b	1.63±0.19 b	33^ b^

All data are given as mean ±SEM, (n=8).

METH: Methamphetamine; Li: Lithium.

a *P*<0.001*vs *control group.

b *P*<0.001*vs *methamphetamine treated group

**Table -2:** Effects of various doses of lithium on methamphetamine-induced oxidative stress in rats

**Groups**	**MDA ** **(** **nmol/mg** ** of** ** protein** **)**	**SOD** **(U/ml/mg of protein)**	**GPx** **(mU /mg of protein)**	**GR** **(mU /mg of protein)**
**Control**	8.7±1.9	81±7.9	139±5	139±7
**METH (10 mg/kg)**	18±2 a	52±6^a^	119±7 a	118±6 a
**METH (10 mg/kg) +Li (75 mg/kg)**	15±1.8	48±5	125±8	126±9
**METH (10 mg/kg) +Li (150 mg/kg)**	11±1.2^b^	71±5^b^	131±6^b^	132±8^b^
**METH (10 mg/kg) +Li (300 mg/kg)**	10±1.1 b	75±6^b^	132±8 b	133±7 b

All data are given as mean ±SEM, (n=8).

METH: Methamphetamine; Li: Lithium.

a *P*<0.001*vs *control group.

b *P*<0.001*vs *methamphetamine-treated group

*A) Handling*

Before the experiment and on the first day, for adaption of animals, all rats were located in the tank filled with 40 ^°^C water (room temperature was 25±2 ^°^C), and the experimenter (blind) directed the rat to swim to the quadrant containing the stand. Throughout the experiment, the stand was placed in the tank, South-East quarter (25, 26).

*B) Training procedure*

In order to facilitate learning of stand’s position by animals, some external cues (for example a distinguishing window, picture, or door) were placed, as spatial cues, in the extra maze. The stand was placed in the South-East quarter of the MWM tank, which was located at a distance of 25 cm from the edge of the tank, and 1 cm beneath the water surface. Four trials per day during four consecutive days were performed on each rat for assessing the learning process. Each rat was randomly placed in one of the mentioned quadrants (North, East, West, and South). During learning time, if the stand was found by the rats within 60 sec, the trial was terminated automatically by the computer, but if they failed to find the stand within 60 sec, the trial was stopped by computer. For measuring learning time, three parameters were evaluated:

 1. The time of escape latency, which is characterized by the time to find the hidden stand by the rat 

 2. The distance traveled by each rat to reach the hidden stand.

 3. Speed of each rat to reach the hidden stand.

*C) Memory evaluation procedure*

The procedure was done on the probe day (fifth day) where the stand was removed, and the animal was in the memory assessment phase. In this day animalss‏ were randomly frightened of the water from any of the directions mentioned above (almost East). Presence percentage of animals in the target quarter (South-East quarter in our experimental) was recorded and calculated (25-29).

***Molecular assessment***

*Mitochondrial preparations*

By administration of 50 mg/kg sodium thiopental, IP, all animals were anesthetized, and the total hippocampus tissue was detached from each rat. After homogenization in cold buffer [25 mM 4-morpholinepropanesulfonic acid, 400 mM sucrose, 4 mM magnesium chloride (MgCl_2_), 0.05 mM ethylene glycol tetraacetic acid (EGTA), pH 7.3] the tissue was centrifuged at 450 × *g *for 10 min. Next, its supernatants were re-centrifuged at 12000 × *g* for 10 min. Lastly, the sediments were re-suspended in the stated homogenization buffer and kept at 0 °C. The amount of total mitochondrial proteins in tissues was determined, according to the guideline of DC protein assay commercial kit (Bio-Rad), (California, USA). For this valuation; Bradford reagent (1 part Bradford: 4 parts dH_2_O) was added serial dilutions (0.1-1.0 mg/ml) of a known protein sample concentration, e.g., bovine serum albumin (BSA) dissolved in homogenization buffer. These sequences of serial dilutions were used to generate a standard curve. Different quantities of 10, 15, 20, 25, and 30 μl of the protein extract (homogenized tissue solutions) were added to several wells. Bradford reagent was also added to each of them. Wells were read by the plate reader at 630 nm, to measure the density of colors. Using various BSA concentrations, the standard curve was drawn and used for measurement of the protein present in the extract. The homogenized solution, comprising mitochondria of the hippocampal cell tissue, was studied for evaluation of apoptosis, inflammatory biomarkers, and oxidative stress (17, 30).

***Measurement of oxidative stress biological markers***

*Evaluation of lipid peroxidation*

In order to evaluate lipid peroxidation, malondialdehyde (MDA), the main biomarker of lipid peroxidation was measured. Totally 100 μl of the lysis solution, SDS, was added to tubes that contained the sample solution (100 μl) or MDA standard. After shaking and incubation, 250 μl of thiobarbituric acid (TBA) substance was added to each tube and incubated at 95 °C for 45–60 min. Afterward, the tubes were centrifuged at 1000 × g for 15 min, and 300 μl of n-butanol was added to 300 μl of the supernatant and then centrifuged for 5 min at 10,000 × g. Lastly, the absorbance of each tube was read at 532 nm, and the outcomes were expressed as nmol/mg of protein (18, 31-35). 

***Measurement of GSH (Glutathione) and GSSG (Glutathione disulfide) disulfide***

To measure GSSG (glutathione disulfide) and GSH (glutathione) levels, 25 μl of the 1X glutathione reductase solution and 25 μl of the 1X NADPH solution were added to a 96-well plate comprising a standard solution of glutathione or mentioned homogenized sample solution. Next, 50 μl of the IX chromogen was added to each well and mixed robustly. To end, the absorbance was read at 405 nm for each GSSG/GSH standard and sample. Quantities of GSSG/GSH were measured and reported as nmol/mg of protein, using the standard curve (18, 36). 

***Measurement of manganese superoxide dismutase (MnSOD) function***

SOD activity was measured conferring to the previously described method (18, 36, 37), and was measured by the following standard equation: SOD activity = {[(A blank 1 - A blank 3) – (A sample - A blank 2)]/ (A blank 1 - A blank 3)} ×100.

***Measurement of glutathione peroxidase (GPx) function***

GPx activity was measured according to previous methods (18, 36, 37). It was measured according to absorbance change [ΔA340/min] and detected by the following equation: ΔA340/min= A340 nm (Start) – A340 nm (Stop)/ Reaction time (min), any alteration in the absorbance is directly related to GPx activity. 

GPx activity: ΔA340/min × Reaction volume (ml) × Dilution factor of the original sample / Extinction coefficient for NADPH at 340 nm× Volumes of the tested sample. Outcomes were described as mU /mg protein (18, 36, 37).

*Evaluation of glutathione reductase (GR) function*

GR function was measured as described previously (18, 36, 37) and its detection was based on the change in absorbance [ΔA340/min] by the subsequent equation: ΔA340/min= A340 nm (Start) – A340 nm (Stop)/ Reaction time (min), any variation in the absorbance is directly related to GR activity. GR activity: ΔA340/min × Reaction volume (ml) × Dilution factor of the original sample / Extinction coefficient for NADPH at 340 nm× Volumes of the tested sample. Outcomes were stated as mU/mg protein (18, 36, 37).

*Evaluation of protein expression/level alteration*

Concentrations (expression or level of protein) of cyclic adenosine monophosphate (cAMP) Response Element Binding Protein (CREB) (total and phosphorylated), brain-derived neurotrophic factor (BDNF), GSK3 (total and phosphorylated), Akt-1(total and phosphorylated), TNF-α, IL-1β, Bcl-2, and Bax in cell lysate of hippocampal tissue, were measured by ELISA kits (Genzyme Diagnostics, Cambridge, U.S.A). Concisely, first, wells containing sheep anti-rat BDNF,CREB (total and phosphorylated), IL-1β, and TNF-α polyclonal antibody (Sigma Chemical Co., Poole and Dorset, UK) were washed three times with washing buffer [containing 0.5 M Sodium chloride (NaCl), 2.5 mM sodium dihydrogen phosphate (NaH_2_PO_4_), 7.5 mM Na_2_HPO_4_, 0.1% Tween 20, pH 7.2]. Then, 100 ml of 1% (w/v) ovalbumin (Sigma Chemical Co, Poole, Dorset, UK) solution was added to each well and incubated at 37 ^°^C for 1 hr. After that following three washes, 100 ml of samples and standards were added to each well and incubated at 48 ^°^C for 20 hr. Again After three washes, 100 ml of the biotinylated sheep anti-rat IL-1β or TNF-α antibody (1:1000 dilutions in washing buffer containing 1% sheep serum, Sigma Chemical Co, Poole, Dorset, UK) was added to each well. Next, after 1-hr incubation and three washes, 100 ml avidin-HRP (Dako Ltd, UK) (1:5000 dilution in wash buffer) was added to each well and the plate was incubated for 15 min. After washing three times, 100 ml of TMB substrate solution (Dako Ltd., UK) was added to each well and then incubated for 10 min at room temperature. Then, 100 ml of 1M H_2_SO_4_ was added, and absorbance was read at 450 nm. Results of TNF-α, IL-1β, Bax, and Bcl-2 in hippocampus tissues were reported as ng/ml, and CREB (total and phosphorylated), BDNF, Akt-1(total and phosphorylated), and GSK3 (total and phosphorylated) results were reported as pg/ml (38-41).

***Statistical investigation***

Data were evaluated using the GraphPad PRISM Software (version 6). Data were expressed as the mean ± standard error of the mean (SEM). Treatment and control group differences were first evaluated by ANOVA and followed by *post hoc *Tukey’s test. A *P*-value<0.001 was considered statistically significant. The analysis was performed by a statistician blinded to the experimental groups. 

## Results

***Results of changes in escape latency and distance traveled through training day in MWM***

Compared to control groups (*P*<0.001), METH (10 mg/kg) caused increased parameters of escape latency and traveled distance through the four days of training and learning in the MWM and this change was remarkable (Figures 1A and B). In contrast, lithium (150 and 300 mg/kg) repressed METH-stimulated significant reduction in escape latency and distance traveled (*P*<0.001) (Figures 1A and B).

***Results of changes in swimming speed through training days***

During training trials, the swimming speed was comparable between the experimental groups (Figure 1C).

***Results of percentage in the target quadrant in the probe trial***

In comparison with the control group, METH (10 mg/kg), caused a noteworthy reduction in animals’ presence percentage, in the target quarter (*P*<0.001) (Figure 1D). Also, lithium (150 and 300 mg/kg) can significantly diminish METH-induced reduction in animals’ presence in the target quarter (*P*<0.001) (Figure 1D).

***Effects of various doses of lithium on METH-induced GSH/GSSG variations***

METH (10 mg/kg) treatment significantly decreased the mitochondrial GSH content while increasing the GSSG levels compared to the control group (*P*<0.001) (Table 1). On the other hand, an increase in GSH-content and significantly reduced GSSG levels in METH-treated animals, in lithium at high doses (150 and 300 mg/kg) groups, was seen in comparison to the methamphetamine only treated group (*P*<0.001) (Table 1). 

***Effects of various doses of lithium on METH-induced variation in oxidative stress parameters***

There was a significant increase in MDA levels and decreased SOD, GPx, and GR activity after METH administration, compared to the control group (*P*<0.001) (Table 2). On the other hand**, **high doses of lithium (150 and 30 mg/kg) attenuated METH-induced increase in MDA levels and reduced SOD, GPx, and GR activity (*P*<0.001) (Table 2).

***Effects of various doses of lithium on METH-stimulated rise in inflammatory biomarkers***

METH, 10 mg/kg, caused a profound increase in levels of IL-1β and TNF-α when compared to the control group (*P*<0.001) (Figures 2 A and B). When compared to METH only treated group (*P*<0.001) high doses of lithium (150 and 300 mg/kg) prevented these METH-induced rises in IL-1β and TNF-α levels, which is a significant change (Figures 2 A and B).

***Effects of various doses of lithium on METH-stimulated changes in Bax and Bcl-2 protein levels***

There was increased Bax protein levels and attenuated Bcl-2 protein levels after METH (10 mg/kg) treatment when compared to the control group (*P*<0.001). In contrast, high doses of lithium (150 and 300 mg/kg) increased Bcl-2 while reducing Bax levels when compared to the METH only treated group (*P*<0.001) (Figures 3 A and B).

***Effects of various doses of lithium on METH-stimulated alteration in protein expression/level of both forms of CREB and BDNF***

METH (10 mg/kg) administration significantly decreased the protein expression/level of BDNF and CREB (total and phosphorylated) when compared to control group (*P*<0.001) (Figures 3 C, D, and E). Conversely, in comparison to the METH only treated group (*P*<0.001), there was a significant change at high doses of lithium (150 and 300 mg/kg) in METH-dependent animals, which led to increase in protein expression/level of BDNF and CREB (total and phosphorylated) (Figures 3 C, D, and E).

***Effects of various doses of lithium on METH-stimulated alteration in protein expression/level of both forms of Akt-1 and GSK3***

METH (10 mg/kg) administration markedly reduced the protein expression/level of Akt-1 (total and phosphorylated) and increased GSK3 (total and phosphorylated) when compared to the control group (*P*<0.001) (Figures 3 F, G, H, and I). After administration of high doses of lithium (150 and 300 mg/kg), in METH-dependent animals, there was a significant improvement in the protein expression of Akt-1(total and phosphorylated) and reduction in GSK3 (total and phosphorylated), when compared to the METH only treated group (*P*<0.001) (Figures 3 F, G, H, and I).

## Discussion

Results show that lithium at various doses ameliorates METH-stimulated oxidative stress, neuro-apoptosis, and inflammation in the rat hippocampus. We also observed that the protecting effects of lithium were intermediated at least in part through P-CREB /BDNF and Akt-1 /GSK3 signaling pathways. METH as a psycho-stimulant has a high potential for misuse and addiction (9, 42). The current study shows that prolonged administration of METH at a dose of 10 mg/kg can increase escape latency and distance traveled in MWM, suggestive of METH-induced learning deficits. Furthermore, in probe day, METH administration reduced the time percentage spent in the target quarter in MWM, suggestive of compromised spatial memory.

Our results approve the previous findings concerning METH-induced deficits in learning and memory in rats (9, 43). It has been shown that the release of dopamine, serotonin, and adrenaline in the brain has been stimulated by METH, which causes down-regulation of these amine receptors following cognitive impairment (43, 44). According to our results, lithium at high doses (40 and 80 mg/kg) reduced METH-stimulated cognition impairment. This protective influence of lithium on memory and learning has also been presented by previous studies (45, 46).

Our data indicated that administration of METH increased hippocampal MDA levels, whereas, lithium treatment (75, 150, and 200 mg/kg) attenuated METH-stimulated rise in lipid peroxidation in the brain. At higher doses (150 and 300 mg/kg) the suppressive influence of lithium on MDA levels was more profound compared to lower doses (75 mg/kg), used in this study. These outcomes are similar to previous findings, which have also shown METH-stimulated lipid peroxidation in the brain (9, 47). The adverse effects of METH are potentially mediated by its effect on mitochondria, and that lithium has the potential to reduce METH-induced toxicity, these results are confirmed by our studies and previous findings (48, 49). Moreover, it is well-evident that lithium has a role as a scavenger for free radicals in neurodegenerative diseases such as Alzheimer’s (49). 

The results from this study indicated that METH (10 mg/kg) causes mitochondrial GSH decline while increasing GSSG levels in the hippocampal tissues. A key change that can promote neurodegenerative signals in the brain is activation of glutathione reduced form (GSH) to the toxic oxidized form (GSSG) by METH (50-53), and this mechanism adversely affects glutathione cycle and leads to neural cell death (51, 53). Furthermore, we found that various doses of lithium, particularly 150 and 300 mg/kg, increase GSH content while decreasing GSSG level in animals with METH (10 mg/kg) abuse. According to studies, neuroprotective effects of lithium against neurodegenerative disorders is mediated via modulation of the glutathione circle (54, 55). 

In our study, administration of METH decreased GPx, GR, and SOD activities in isolated hippocampal tissues. Studies have also reported a reduction of antioxidant defenses with METH, which may result in neurodegeneration (43, 56). It has been shown that for transforming the oxidized form of glutathione (GSH) to the reduced form (GSSG), GR is the key enzyme (57). Consequently, METH-induced reduction in GR function results in an increase of GSSG and decrease of GSH levels as witnessed in this study. Several new reports presented that METH causes mitochondrial dysfunction that prompts antioxidant enzyme activity inhibition in multiple cells (9, 30, 43). We detected that lithium treatment helps in recovering the action of antioxidant enzymes in a dose-dependent manner. Lithium protects the brain against METH-stimulated oxidative stress by triggering GR, which increases the transformation of GSSG to GSH. The anti-oxidative properties of lithium in neurodegenerative disorders are well-known according to prior experimental studies (58, 59). Along with previous reports, our results confirmed METH-induced reduction in SOD activity (60). Conversely, lithium treatment elevated SOD activity thereby defending the tissue against oxidative impairment. Formerly, it was discovered that lithium was advantageous in reversing alcohol abuse-related decrease in SOD activity in the hippocampal tissues (54). We demonstrated that, in the hippocampal tissue, chronic METH administration profoundly induces the levels of pro-inflammatory cytokines like TNF-α and IL-β. However, lithium at high doses is likely to suppress METH-stimulated neuroinflammation. Our results approve previous reports regarding METH and other psychostimulants-induced increase in pro-inflammatory cytokines. It has been suggested that pro-inflammatory action of METH is accountable for its neurodegenerative properties (61). Conversely, lithium has been shown to target neuroinflammation signaling cascades, thus maintaining the brain against inflammation and related impairment (20).

Other than oxidative stress and inflammation, this study approves METH-induced apoptosis in the hippocampus. We witnessed that METH administration results in raised Bax apoptotic protein; however, it declines Bcl-2 anti-apoptotic protein. This data is in line with prior works that have demonstrated that METH misuse can cause brain impairment through activation of multiple apoptotic cascades (62, 63). In contrast, our study outcomes confirmed the anti-apoptotic influence of lithium against METH administration, as shown by reduced Bax and enhanced Bcl-2 expressions in the hippocampus. Earlier studies revealed that lithium treatment could reduce levels of cleaved caspase-3 and Bax and subsequent nuclear condensation that leads to neurodegeneration (16).

Previously, the anti-inflammatory, anti-apoptotic, and anti-oxidative properties of lithium have been described (20, 59); however, the involved signaling pathways persist unidentified. Concerning this, we assessed the role of the Akt-1/GSK3 and P-CREB-BDNF signaling pathways. According to our data METH administration decreases BDNF, CREB (total and phosphorylated), Akt-1 protein expression, and GSK3 level in the hippocampus. In contrast, lithium at high doses enhanced CREB (total and phosphorylated), BDNF and Akt-1 expression and GSK3 levels. Therefore, it can be predicted that treatment with lithium reestablishes the P-CREB-BDNF signaling cascade and keeps the brain against METH-simulated neurotoxicity. The P-CREB, as a transcription factor, adjusts over hundred genes, mainly BDNF which is strongly associated in neuronal regeneration, development, survival, excitability, addiction, depression, and cognition (30, 64). Additionally, it has been shown that dysregulation of CREB transcriptional cascade prompts oxidative stress, apoptosis, and neurodegeneration (64, 65). Several studies have stated that P-CREB (activated form of CREB) assists the expression of BDNF, a TrkB receptor ligand. Furthermore, stimulation of the TrkB receptor by BDNF prevents brain cell deterioration and progress endurance of neurons (66). This cascade signaling including P-CREB-BDNF has also been linked to cognitive functions, mood balances, and reward mechanisms (66-69). In the present study, METH reduced activity of BDNF/TrkB signaling pathway and stimulates neurodegeneration, apoptosis, inflammation, and oxidative stress. In contrast, there seems to be an improvement in P-CREB/BDNF/ TrkB signaling with lithium administration, thus protecting the hippocampus against METH-induced toxic harm. Glycogen synthase kinase-3 (GSK-3) dysfunction is associated with numerous brain diseases, mainly with neuronal apoptosis, neurodegeneration, and cognitive damage. Stimulation of phosphatidylinositol 3-kinase (PI3K) and following phosphorylation of Akt-1 (Protein Kinase B) inhibits GSK-B. Additionally, inhibition of GSK-3 is related to neuronal survival and mood stabilization. In the current study, we witnessed an increase in GSK3 and reduction in AKT-1 expression by METH abuse. On the contrary, lithium-treated animals revealed improved AKT-1 expression and lower expression of GSK3.

## Conclusion

Taken together, for the first time, the outcomes of the present study display that lithium treatment, probably through stimulation of P-CREB-BDNF and Akt-1/GSK3 signaling pathways, can attenuate METH-induced apoptosis, inflammation, and oxidative stress. Though, additional studies concerning toxicity and human dosage are required.

## Coflicts of Interest

None to declare.
